# Human-specific genetic hallmarks in neocortical development: focus on neural progenitors

**DOI:** 10.1016/j.gde.2024.102267

**Published:** 2024-10-08

**Authors:** Lidiia Tynianskaia, Michael Heide

**Affiliations:** German Primate Center, Leibniz Institute for Primate Research, Kellnerweg 4, 37077 Göttingen, Germany

**Keywords:** neocortex development, neocortex evolution, human-specific genes, neural progenitors, modern human-specific changes

## Abstract

The evolutionary expansion of the neocortex in the ape lineage is the basis for the development of higher cognitive abilities. However, the human brain has uniquely increased in size and degree of folding, forming an essential foundation for advanced cognitive functions. This raises the question: what factors distinguish humans from our closest living primate relatives, such as chimpanzees and bonobos, which exhibit comparatively constrained cognitive capabilities? In this review, we focus on recent studies examining (modern) human-specific genetic traits that influence neural progenitor cells, whose behavior and activity are crucial for shaping cortical morphology. We emphasize the role of human-specific genetic modifications in signaling pathways that enhance the abundance of apical and basal progenitors, as well as the importance of basal progenitor metabolism in their proliferation in human. Additionally, we discuss how changes in neuron morphology contribute to the evolution of human cognition and provide our perspective on future directions in the field.

## Introduction

The neocortex is a six-layered brain structure which evolved around 220 million years ago exclusively in the mammalian lineage^1–3^. Its emergence has been associated with the evolution of intricate perceptual, cognitive, emotional, and motor skills^4,5^. In primates, the evolutionary expansion of the neocortex resulted in an exceptionally high number of neurons and an increased degree of folding (gyrification), with humans surpassing other apes in these characteristics. For instance, the human neocortex harbors approximately 16 billion neurons, whereas our closest living relative, the chimpanzee, possesses only around 7.4 billion neurons^6,7^. Although the number of cortical neurons is one fundamental basis for human intelligence, it is by no means the only determining factor, with other important elements including interneuronal distance and high conduction velocity^8–10^.

The intriguing question of which genetic changes contributed to the emergence of human-specific neurodevelopmental traits has gained significant attention in recent years. These genetic changes can range from single point mutations to the generation of new genes. New genes always evolve from sequences that are already present in the genome using multiple mechanisms. These mechanisms for gene evolution include gene duplication, fusion, alternative splicing and *de novo* gene birth^10–12^. *De novo* genes, which evolve from non-coding DNA sequences that acquire functionality through mutations generating or restoring an open reading frame, are currently particularly understudied in the field^13,14^. The question arises, through which mechanisms would these genetic changes translate into massive interspecies differences in brain mass and folding? Functionally, this is believed to primarily be achieved through the modification of the activity and the behavior of neural progenitor cells (NPCs), which produce the majority of cortical neurons and shape cortical morphology^15–17^.

NPCs can be classified into apical and basal progenitors (APs and BPs). APs are the primary progenitor type giving rise to all further progenitor and neuron types. They reside in the ventricular zone (VZ), the primary germinal zone of the developing cortical wall directly lining the ventricle. As these progenitors can only divide at the ventricular surface, the number of their mitotic divisions is limited due to spatial restrictions^18–20^. This hinders the maximization of cortical expansion, which, during evolution, resulted in the formation of a second germinal zone, the so-called subventricular zone (SVZ), basal to the VZ. The SVZ is populated by BPs that are initially generated by AP divisions and can subsequently divide freely within the SVZ. Therefore, the BPs are thought to be key for neocortex expansion. Especially one type of BPs with high proliferative capacity – referred to as the basal or outer radial glia (bRG or oRG) – seems to be a key factor for the large and strongly folded human neocortex^18–21^. In contrast to bRG, the second type of BPs, known as basal intermediate progenitors (bIPs), are less proliferative and are the predominant BP in lissencephalic species such as rodents^18,22,23^. The high proliferation rate of BPs (mainly bRGs) significantly expands the progenitor pool and consequently the number of neurons produced^4,18^.

**Figure 1:**
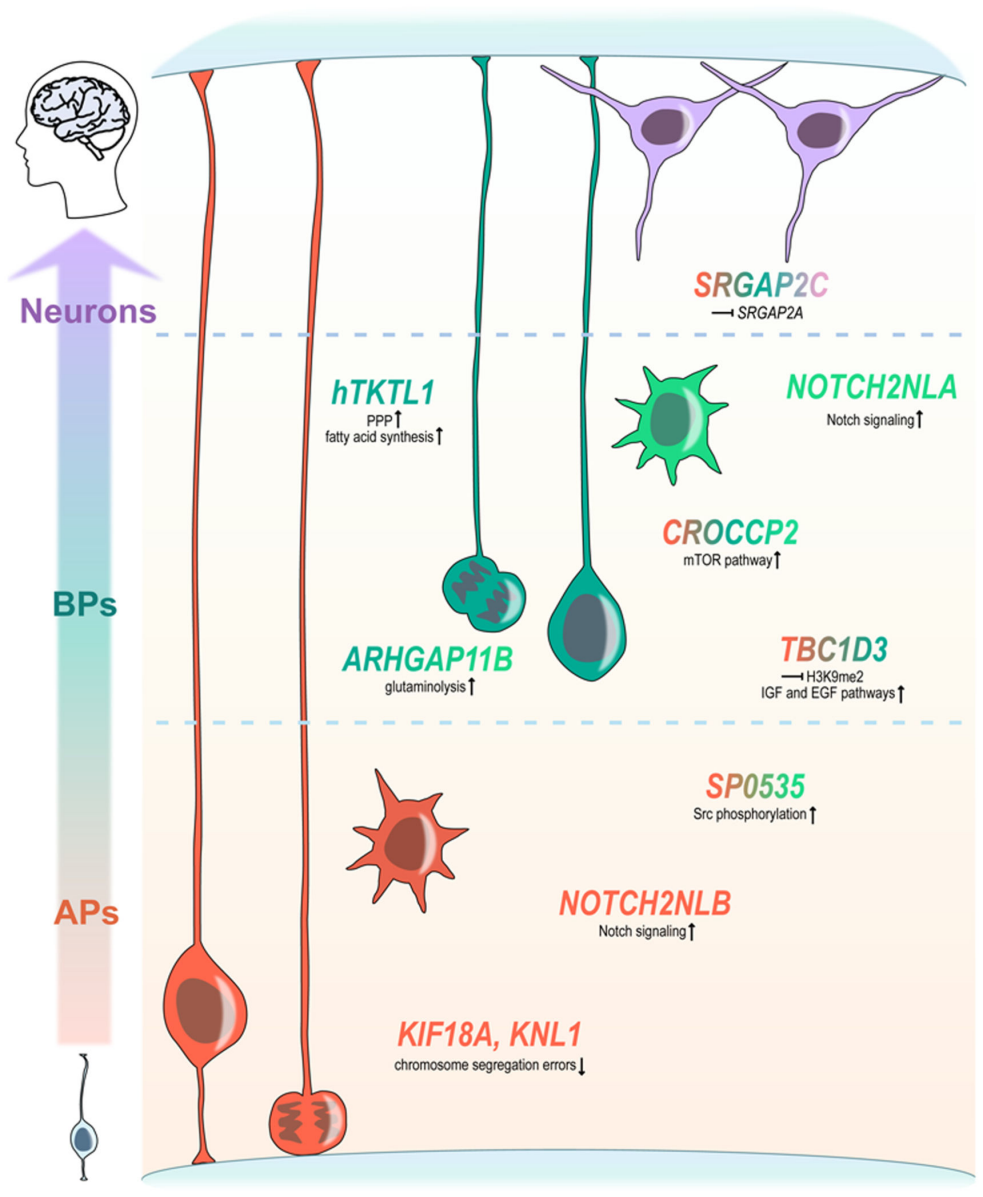
A graphical representation of genes with human-specific characteristics and their associated cell types in neurodevelopment. Gene names are color-coded to represent the cell type(s) they affect: apical progenitors – orange, basal progenitors – green, neurons – light violet. A short description of the molecular mechanism of action for each gene is provided in black text below.

The question of human-specific genetic changes contributing to our cognitive abilities has previously gained significant attention in the field. One well-known example is *FOXP2*. Human *FOXP2* underwent positive selection in the course of human evolution and was found to play a role in neurogenesis regulation, neurite outgrowth and circuit formation^24,25^. Despite the critical contributions of this earlier research, in this review, we will concentrate on discussing recent studies from the past two years that investigated (modern) human-specific genetic changes affecting predominantly NPC behavior and activity (summarized in Table 1 and Figure 1).

### Signaling pathway modulation and quality control in human apical progenitors

Conceptually, to increase the processing power of the brain, neurogenesis and neural maturation can be influenced at multiple levels, from APs to neurons. In the course of this review, we will follow this lineage starting with the effects on APs. As APs are the primary source of the neural lineage, increasing their pool size would result in an expansion of all subsequent cell types. From the evolutionary perspective, this would translate into an elevated neuron number and potentially an increased brain size. Indeed, a multiplicity of human-specific genes was previously found to enhance the number of proliferative APs^26,27^. A typical way to increase proliferation is to modify signaling pathways. One example is the human-specific gene *NOTCH2NLB* which activates Notch signaling to promote AP self-amplification and to delay neuronal differentiation^27,28^. Here we present recent cases of human-specific genes, which modify an existing signaling pathway relying on mTOR and Src signaling leading to enhanced proliferation.

The human-specific *de novo* gene *SP0535* was shown to enhance AP cell proliferation via elevating Src phosphorylation levels. *SP0535* has evolved by a two-nucleotide deletion creating a novel open reading frame resulting in a 103-amino acid long protein^29^. Functional analysis of human *SP0535*-KO cortical organoids and *SP0535*-transgenic mice revealed that this gene promotes increased proliferation of APs and elevates bIP numbers. Consequently, this results in more neurons, along with expansion and folding of the mouse cortex. Moreover, these mice display improved cognitive skills and working memory. These functions are mediated by incorporation of SP0535 into an existing molecular network through releasing Src from ATP1A1-Src complex and its subsequent phosphorylation leading to cell proliferation^30^.

A further gene elevating proliferative AP level via modifying a signaling pathway is *CROCCP2*. It most likely arose within the Catarrhini (old-world monkeys) lineage via partial duplication of its ancestral gene, *ciliary rootlet coiled coil* (*CROCC*), which encodes Rootletin, a protein constituting the ciliary rootlet^31^. In a recent publication, *CROCCP2* was proposed to be exclusively expressed in the human fetal cortex compared to other apes^32^. Its introduction into the fetal mouse brain led to an increase in proliferative APs and bIPs accompanied by successive elevation of neuron numbers via activation of indirect neurogenesis. Interestingly, *CROCCP2* knock-down (KD) in human cerebral organoids reduces both bIP and bRG levels while increasing neuron numbers, likely due to APs switching to direct neurogenesis. The CROCCP2-mediated effect was found to be conveyed by its inhibitory interaction with the ciliary trafficking protein IFT20, causing a decreased ciliary length. This then activates mTOR signaling which was shown to be upregulated in progenitors during human corticogenesis^33–36^. Ultimately, this suggests a link between mTOR signaling and ciliary dynamics modulated by *CROCCP2*.

While the majority of studies focus on AP proliferation, recently genomic integrity came into focus as well. As mitosis is repeatedly executed in proliferating cells, the statistical chance of progeny inheriting an incorrect chromosome number increases. Since APs divide frequently in order to serve as the primary source for the whole neural lineage of the cortex, it is critical to maintain genomic integrity in these cells. Consequently, in species where APs typically generate more progeny, enhanced quality control mechanisms during mitosis are likely required. In fact, modern human-specific variants of KIF18 and KNL1 proteins were discovered to contribute to accurate mitotic chromosome segregation. This effect is achieved due to their localization at the kinetochore, where these genes control the initiation of chromosome segregation upon their proper alignment at the metaphase plate^37^. *KIF18a* and *KNL1* were found to each have one and two amino acid substitutions unique to modern humans compared to archaic ones, respectively^38^. Upon “modern humanization” of the corresponding genes in mouse, they were found to have an additive effect on prolonging metaphase, consequently reducing the number of lagging chromosomes. On the contrary, APs in “ancestralized” for *KIF18* and *KNL1* human cerebral organoids showed shorter metaphase and more lagging chromosomes than modern human control. Therefore, the emergence of modern human-specific amino acid substitutions in kinetochore-associated proteins KIF18 and KNL1 led to improved chromosome segregation fidelity by extending the duration of metaphase^38^.

So far, research has predominantly focused on identifying factors that influence the proliferative capacity of APs and ultimately cause an increase in progenitor numbers. However, ensuring the genomic integrity of these progenies is equally important. To achieve this, the quality control mechanisms during mitosis must be fine-tuned to prevent genetic defects, such as chromosomal segregation errors. Future research should explore factors that enhance division control mechanisms, which likely exist not only at the chromosomal level but also for subtler mutations during other phases of the cell cycle (like replication errors during s-phase). Given the fundamental importance of improving such control mechanisms, it would be interesting to explore the possibility of positive selection for them in species with expanded AP lineages like other ape species.

### Signaling and metabolic regulation in human basal progenitors

After expanding their pool through symmetrical divisions, APs switch to BP production. The expansion of BPs is crucial for significantly increasing the final output of neurons. Similar to APs, increased BP proliferation can be achieved by modifying signaling pathways. A well-established human-specific gene acting in this manner is *NOTCH2NLA*, which promotes bIP amplification through NOTCH signaling (ref). Recently, additional examples of human-specific genes activating various signaling pathways to promote BP proliferation have been described. This also includes *CROCCP2* and *SP0535*, discussed in detail above, as both increased the number of bIPs, in addition to expanding the AP pool, in fetal transgenic (TG) mouse brain^29,32^.

Another interesting instance is the *TBC1D3* gene family which emerged in the Simian infraorder through large-scale chromosomal rearrangements and duplication events with varying evolutionary trajectories and copy numbers across different primate lineages. In human, only a selected few copies appear to be actively expressed, each of which harbors a distinct human-specific carboxy-terminal sequence^39^. Mouse *in utero* electroporation and KD experiments in human fetal brain slices showed that *TBC1D3* promotes aRG delamination and bRG proliferation, as well as cortical folding induction in some mice^40^. A further study conducted on human cerebral organoids postulated that the molecular mechanism of *TBC1D3*-induced progenitor amplification relies on the inhibition of G9a-mediated H3K9me2 modification. This epigenetic mark is known for its suppressive role in regulating proliferative gene expression^41^. Another previously proposed theory suggests that the impact of *TBC1D3* on cell proliferation is conveyed via the insulin-like growth factor-1 (IGF) and epidermal growth factor (EGF) pathways^42,43^.

Besides signaling cascades, another major modifier of proliferation that recently emerged is metabolism^44^. Indeed, one of the most well-studied human-specific genes, *ARHGAP11B*, is involved in BP metabolism. *ARHGAP11B* evolved by partial duplication from *ARHGAP11A* with a subsequent point mutation generating a new splice donor site resulting in a novel human-specific C-Terminus ^45–48^. Interestingly, in contrast to *ARHGAP11A*, which localizes to the nucleus, *ARHGAP11B* is imported into mitochondria. There, it interacts with adenine nucleotide translocase (ANT) to block the mitochondrial permeability transition pore (mPTP), resulting in increased mitochondrial Ca^2+^ concentration and stimulation of glutaminolysis^49^. This allows *ARHGAP11B* to increase bRG abundance, which elevates neuron numbers and can induce gyrification^46,50–53^. A recent study has further confirmed *ARHGAP11*’s role in increasing bRG numbers by introducing it into chimpanzee cerebral organoids. Moreover, rescue experiments in the form of *ARHGAP11B* electroporation in *ARHGAP11A* plus *ARHGAP11B* double KO human organoids showed that *ARHGAP11B* is necessary for increased proliferation of bRG during human cortical development^54^. Another recent publication showed that the combined expression of *ARHGAP11B* and of the ape-specific glutamate dehydrogenase 2 (*GLUD2)* in the embryonic mouse neocortex can enhance *ARHGAP11B*-induced bRG abundance but not that of bIPs^55^. It appears that the evolutionary emergence of *ARHGAP11B* enhanced the metabolic pathway linked to *GLUD2*, increasing alpha-ketoglutarate and subsequent aspartate production essential for *de novo* nucleotide synthesis in bRGs. This suggests that metabolic processes can be modified throughout evolution, for example, by means of developing functional synergies, to maximize progenitor proliferation.

Transketolase-like 1 (TKTL1), a further gene specifically implicated in human bRG proliferation by metabolism activation, carries a single modern-human specific mutation compared to its archaic counterpart. This seemingly minor variation was demonstrated to increase bRG abundance and, consequently, neuron numbers upon introduction into mouse and ferret embryonic neocortex, while reverting *hTKTL1* to its ancestral state in human cerebral organoids resulted in the opposite effect^56^. Moreover, *hTKTL1* induced the formation of bRG with an increased number of processes in ferret ^56^, which is associated with enhanced proliferative capacity ^57^. These effects are thought to be due to TKTL1’s role in the pentose phosphate pathway and fatty acid synthesis, which provides membrane components to the cell.

While enhancing proliferation through human-specific modifications of signaling cascades is well-established, recent focus has shifted to the role of metabolism in this process. This raises questions about how human-specific genes integrate in these metabolic pathways and whether certain adaptions support their integration. As described above, non-human-specific genes might work in synergy with human-specific factors to enhance progenitor proliferative capacity. This indicates that other genes evolved within the primate lineage may equally contribute to the evolution and development of the human brain. Identifying these genes that integrate human-specific genes in metabolic pathways and possibly other cellular processes might be a formidable task for future studies, just as investigating the role of primate-specific factors as prerequisites for human cortical evolution.

### Morphology changes in human neurons

After the expansion of the progenitor pool and ensuring its genetic integrity, a higher number of neurons is generated by the expanded BP population in human. However, this alone does not fully account for our higher cognitive abilities. Further characteristics of neurons, such as their morphology, contribute to the enhanced processing power of the human brain.

A well-established human-specific gene *SRGAP2C* evolved through incomplete duplication of *SRGAP2A* and conveys its neurodevelopmental effect through functional antagonism of the latter^10,58^. *SRGAP2C* has been shown to cause neoteny of spine and synapse development, along with synaptic density increase^59,60^ affecting connectivity and cognitive abilities^61^. In a recent study, *SRGAP2C* TG cynomolgus macaques showed a temporary increase in neuron numbers which could be traced back to increased progenitor proliferation and, in accordance with previous mouse studies, cell migration^62^. Consistent with observations in mice, *SRGAP2C* TG macaques exhibited neotenic spinogenesis and, additionally, myelination was delayed^60^. Furthermore, these primates demonstrated a general delay and prolongation of brain development, accompanied by region-specific changes in volume, along with enhanced motor planning and execution skills.

Despite some progress in this area, there remains a significant gap in understanding the relationship between the sheer number of cortical neurons and cognitive function. Future research should not only focus on identifying genes that influence neuronal morphology but also on how this morphology translates into functional neural networks. Such studies might benefit from the usage of non-human primate models as shown for *SRGAP2C*.

## Conclusion

Human high cognitive abilities arose from the evolution of numerous factors, including those that caused the expansion of apical and basal progenitor pools, resulting in a higher number of neurons. In this review, we discussed recent findings of (modern) human-specific genetic changes primarily affecting NPC behavior and activity. Interestingly, these changes seem to mainly enhance signaling and metabolic pathways resulting in increased proliferation of NPCs. During human evolution, these pathways were adapted and/or fine-tuned in the pre-existing genetic context. However, the current prevailing strategy in the field is to study each gene in isolation, often using evolutionarily distant from human model organisms. This approach does not accurately reflect the functional networks within which a gene evolved and its potential role within those networks. Future studies need to elucidate combined functional effects of multiple human-specific genetic modifications to gain valuable insights into the true course of evolutionary events. Likewise, the combined effect of human-specific genetic traits and those occurring within the primate clade (like GLUD2, see above) will help us to better understand human neocortical expansion.

Non-human primates and cerebral organoids have proven to be useful tools in this research, as they represent the closest available genetic environment to humans for testing these genes. However, it is important to recognize that considerable differences are present between the genomic contexts of humans and our closest living relatives, chimpanzees, which have likely acquired their own adaptations to control the behavior and activity of NPC. Additionally, accurately modeling the spatio-temporal expression of genes in a model species remains challenging but needs to be addressed in the future.

In conclusion, future studies should aim to elucidate the complete evolutionary picture of a specific feature of the human brain, considering non-human-specific genes as prerequisites, as well as the genetic networks and interactions involved in the establishment of this feature.

## Figures and Tables

**Table 1:** A summary of the evolutionary history, subcellular localization, molecular mechanisms, and neurodevelopmental effects of genes exhibiting human-specific features.

Gene name(s)	Human-specific feature	Evolutionary mechanism/origin	Subcellular compartment	Neurodevelopmental effect	Molecular mechanism
*ARGAPH11B*	Only present in human genome	Segmental duplication of *ARGAPH11A*; novel carboxy-terminal amino acid sequence due to a novel splice donor site	Mitochondrion	Increased BP proliferation, subsequent higher neuron numbers, induction of cortical expansion and folding, improved memory flexibility	Increase in mitochondrial Ca^2+^ concentration leading to enhanced glutaminolysis
*CROCCP2*	Paralogous loci present in the Catarrhini group; Expression during corticogenesis is proposed to be unique to humans	Partial segmental duplication of the ancestral gene CROCC	Cytoplasm (with perinuclear localization, presumably at the Golgi apparatus)	Increased AP and BP proliferation, subsequent increase in neuron numbers	Inhibition of IFT20 resulting into decreased ciliogenesis and activation of mTOR pathway (for BPs)
h*TKTL1*	Present in mammals; modern human-specific version	Modern-human specific substitution of lysine with arginine due to a single nucleotide mutation	Cytoplasm	Increased bRG abundance, subsequent higher neuron numbers	Upregulation of the pentose phosphate pathway and fatty acid synthesis
*KIF18A* and *KNL1*	Widely conserved genes; modern human-specific versions	One and two modern human-specific amino acid substitutions respectively	Kinetochore	Metaphase prolongation and improved chromosome segregation fidelity in APs	Regulation of chromosome positioning and kinetochore microtubule attachment during mitosis
*NOTCH2NLA-C*	Only present in human genome	Multiple segmental duplication rounds of the ancestral *NOTCH2NLR*; Three human-specific paralogous (*NOTCH2NLA, -B, -C*)	Cytoplasm (suggested to be secreted)	Increased AP proliferation, delayed neuronal differentiation (for *NOTCH2NLB*), increased bIP proliferation (for *NOTCH2NLA*)	Activation of Notch signaling
*SP0535*	Only present in human genome	*De novo* origin; single-base mutation and stop codon escape due to a two-base deletion in the human ENST00000370535 locus	Cytoplasm	Increased proliferation of APs, elevated number of bIPs, subsequently higher number of neurons, induction of cortical expansion and folding	Increase of Src phosphorylation (associated with cell proliferation) via releasing it from ATP1A1-Src complex
*SRGAP2C*	Only present in human genome	Incomplete segmental duplication of *SRGAP2A* (its “grandmother”), subsequent duplication of *SRGAP2B* (its “mother”); loss of SH3 and Rho-GAP domains	Cytoplasm (dendrites of cortical neurons)	Increased progenitor proliferation; induction of “human-like” developmental features as sustained radial migration, neoteny of spine and synapse development, delayed myelination, increased spine size and density, increased synaptic density, prolongation of brain development; cognitive improvements	Functional inhibition of the ancestral *SRGAP2A* via physical heterodimerization
*TBC1D3*	Present in Simian genomes but copy number and organization vary; human-specific carboxy-terminal amino acid sequence	Large-scale chromosomal rearrangements and duplication events suggestive of independent expansion in each lineage	Nucleus in oSVZ cells, cytoplasm/membrane in VZ/iSVZ and CP cells	aRG delamination and bRG proliferation, induction of cortical folding	Proposed theories include: (i) Activation of IGF and EGF pathways; (ii) inhibition of G9a-facilitated H3K9me2
